# p53 and Ca^2+^ signaling from the endoplasmic reticulum: partners in anti-cancer therapies

**DOI:** 10.18632/oncoscience.139

**Published:** 2015-03-07

**Authors:** Mart Bittremieux, Geert Bultynck

**Affiliations:** ^1^ KU Leuven, Laboratory of Molecular and Cellular Signaling, Department of Molecular and Cellular Medicine, Campus Gasthuisberg O/N-I bus 802, BE-3000 Leuven, Belgium

**Keywords:** p53, ER-mitochondrial Ca^2+^ signaling, in vivo Ca^2+^ imaging, anti-cancer treatments, cell death

## Abstract

Ca^2+^ transfer from the endoplasmic reticulum (ER) to the mitochondria critically controls cell survival and cell death decisions. Different oncogenes and deregulation of tumor suppressors exploit this mechanism to favor the survival of altered, malignant cells. Two recent studies of the Pinton team revealed a novel, non-transcriptional function of cytosolic p53 in cell death. During cell stress, p53 is recruited to the ER and the ER-mitochondrial contact sites. This results in augmented ER Ca^2+^ levels by enhancing sarco/endoplasmic reticulum Ca^2+^ ATPase (SERCA) activity, ultimately promoting mitochondrial Ca^2+^ overload. The boosting of “toxic” Ca^2+^ signaling by p53 appears to be a critical component of the cell death-inducing properties of chemotherapeutic agents and anti-cancer treatments, like photodynamic stress. Strikingly, the resistance of p53-deficient cancer cells to these treatments could be overcome by facilitating Ca^2+^ transfer between the ER and the mitochondria *via* overexpression of SERCA or of the mitochondrial Ca^2+^ uniporter (MCU). Importantly, these concepts have also been supported by *in vivo* Ca^2+^ measurements in tumor masses in mice. Collectively, these studies link for the first time the major tumor suppressor, p53, to Ca^2+^ signaling in dictating cell-death outcomes and by the success of anti-cancer treatments.

Oncogenes and deregulation of tumor suppressors favor oncogenesis through signaling pathways that impact cell-death resistance, uncontrolled cell proliferation and altered energy production and requirements [1]. During the last decade, alterations in intracellular Ca^2+^ signaling have emerged as an important factor in the development of tumors and their invasive and metastatic properties [2-4]. Cancer cells display alterations in the expression and regulation of different Ca^2+^-transport systems at both the plasma membrane and the membranes of organelles, like the endoplasmic reticulum (ER) and the mitochondria, thereby impacting different hallmarks of cancer progression [3, 5].

Besides remodeling of Ca^2+^-flux pathways at the level of the plasma membrane [2, 6-8], deregulation of Ca^2+^ signaling from the ER, the main intracellular Ca^2+^-storage site, to the mitochondria, the main apoptosis-inducing organelle, serves as an important oncogenic mechanism for driving cancer progression [9]. The ER and the mitochondria are closely connected via contact sites (mitochondria-associated ER membranes, MAMs), containing Ca^2+^-transport systems, including the inositol 1,4,5-trisphosphate receptor (IP R) at the ER and the voltage-dependent anion channel 1 (VDAC1) at the mitochondrial outer membrane [10, 11]. The mitochondrial Ca^2+^ uniporter (MCU) ensures Ca^2+^ transport across the mitochondrial inner membrane into the mitochondrial matrix [12, 13]. MCU mediates the rate-limiting step for the transfer of Ca^2+^ signals from the ER into the mitochondrial matrix.

Hence, Ca^2+^ signals that arise from the ER directly impact mitochondrial processes in a “dual” manner, promoting survival processes like ATP production and basal autophagy as well as cell-death processes like apoptosis [14, 15]. On the one hand, low-level Ca^2+^ signaling (Ca^2+^ oscillations) increases the activity of pyruvate, isocitrate and α-ketoglutarate dehydrogenases and thus drives mitochondrial ATP production and bio-energetics [14]. Interfering with these basal Ca^2+^ fluxes into the mitochondria results in impaired ATP production and in the engagement of AMPK signaling and autophagy as an adaptive survival pathway [16, 17]. On the other hand, excessive Ca^2+^ transients trigger apoptosis by causing the opening of the mitochondrial permeability transition pore [18-20]. This will result in mitochondrial swelling and rupture of the outer mitochondrial membrane, causing the release of pro-apoptotic factors, including cytochrome c, in the cytosol and subsequent activation of caspases and apoptosis [21].

Thus, the impact of oncogenes and tumor suppressors on cell-death resistance may at least in part be mediated by their respective and opposing effects on Ca^2+^ transfer between the ER and the mitochondria [9]. In healthy cells, the proper oncogene/tumor suppressor balance allows for cell survival with an adequate ability to induce cell death in response to various forms of cell stress, thereby preventing the survival of damaged or altered cells (Fig. 1A). There is now a growing list of oncogenes and tumor suppressors that appear to be present at the ER and/or MAMs, thereby directly targeting and controlling IP Rs, VDAC1 and the sarco/endoplasmic reticulum Ca^2+^ ATPase (SERCA), responsible for pumping Ca^2+^ into the ER [9, 22]. The functional impact of oncogenes like Bcl-2 [23-26], Bcl-XL [27, 28] and PKB/Akt [29, 30] is to limit the “cytotoxic” Ca^2+^ transfer from ER to mitochondria, while tumor suppressors like PTEN [31], PML [32], FHIT [33] and BRCA1 [34] promote this “cytotoxic” Ca^2+^ transfer. Furthermore, these proteins can execute their function by a concerted action on different Ca^2+^-transport systems. For instance, Bcl-2 proteins have been reported to suppress excessive Ca^2+^ signaling by i) inhibiting SERCA activity [35] or increasing Ca^2+^ leak from the ER through sensitized IP_3_Rs [25], which both reduce the loading of the ER Ca^2+^ stores, ii) dampening Ca^2+^ release from ER by directly inhibiting IP_3_Rs [36], and iii) limiting mitochondrial Ca^2+^ uptake by inhibiting VDAC1 [26]. Of note, other Bcl-2-family members, like Bcl-XL and Mcl-1, can also favor cell survival by promoting basal pro-survival Ca^2+^ oscillations by enhancing IP_3_R [37, 38] or VDAC1 activity [39, 40]. Indeed, basal VDAC1 activity appears to be essential for the cell growth and proliferation of a variety of cancer cells [41]. Besides oncogenic and tumor suppressor proteins, also microRNAs implicated in cancer can modulate mitochondrial Ca^2+^ uptake [42]. For instance, MCU expression is dynamically controlled by a cancer-related miR-25 [43]. Downregulation of MCU by miR-25 suppresses mitochondrial Ca^2+^ uptake, causing apoptotic resistance and favoring cancer cell survival [43]. In any case, a major impact of the deregulated balance between oncogenes and tumor suppressors in tumor cells is a strong dampening of ER-mitochondrial Ca^2+^ transfers. This will thereby contribute to the excessive cell-death resistance of cancer cells despite on-going pro-death signaling due to oncogenic stress.

**Fig.1:**
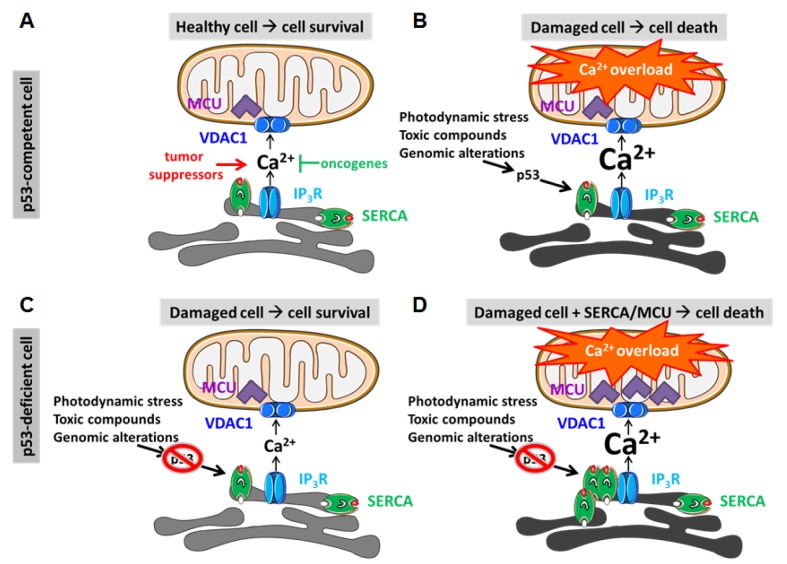
The interplay between p53 and Ca^2+^ signaling for cell death in response to oncogenic stress or anti-cancer treatments *A,* In normal, p53-competent cells (upper row), oncogenes (including PKB/Akt and Bcl-2-family members) and tumor suppressors (PTEN, PML, FHIT, BRCA1 and p53) are in balance, thereby providing the proper flux of Ca^2+^ from the ER into the mitochondria. This delicate ratio allows the production of ATP for survival, while maintaining normal cell death sensitivity. *B,* Upon stress conditions, including photodynamic therapy, toxic compounds or oncogenic stress due to genomic instability and alterations, tumor suppressors, like p53, are activated. This can provoke increased ER-mitochondrial Ca^2+^ transfer, mitochondrial Ca^2+^ overload and the elimination of altered or damaged cells, preventing oncogenesis and cancer initiation/progression. In the presence of p53, SERCA pumps become hyperactive due to p53 recruitment, causing Ca^2+^ overload in the ER and increased sensitivity towards IP_3_ receptor-mediated Ca^2+^ release and subsequent Ca^2+^ uptake into the mitochondria. *C,* In malignant, p53-deficient cells (lower row), excessive cell-death resistance prevails, allowing the survival of damaged or altered cells. These cells fail to engage increased ER-mitochondrial Ca^2+^ fluxes and thus cell death in response to cell stress, toxic compounds or genomic alterations, favoring oncogenesis and cancer progression. These cells are also resistant to anti-cancer therapies, like photodynamic therapy. In part, this is due to their failure to increase SERCA activity due to the absence of p53. *D,* Excitingly, p53-deficient cells can be re-sensitized to photodynamic therapy by promoting ER-mitochondrial Ca^2+^ fluxes *via* overexpression of SERCA, thereby increasing ER Ca^2+^ levels, or *via* overexpression of MCU. These strategies facilitate mitochondrial Ca^2+^ uptake in the mitochondrial matrix, thereby promoting mitochondrial Ca^2+^ overload in response to cell stress.

Yet, until now, the role of Ca^2+^ signaling for the pro-apoptotic function of p53, a major tumor suppressor critical for cell death in response to cell stress and chemo-/phototherapy but mutated in >50% of all human cancers [44], remained elusive. Also, the *in vivo* relevance of Ca2+ signaling from ER to mitochondria for cell-death therapies in cancers and how this is impacted by p53 was not known. Two recent studies by Pinton and co-workers [45, 46] addressed these key points and revealed groundbreaking insights in the p53/Ca^2+^ signaling connection for inducing cell death in cancer cells in response to anti-cancer treatments. These studies reveal a novel, non-transcriptional role for cytosolic p53 at the level of the ER Ca^2+^ stores (Fig. 1B-D). First, Pinton and co-workers demonstrated that photodynamic therapy prominently elevated cytosolic and mitochondrial Ca^2+^ levels in mutant Ras-transformed cells *in vitro*, contributing to the photo-induced stress and cell death. Strikingly, transformed cells lacking p53 displayed a reduced [Ca^2+^] rise from the ER, preventing mitochondrial Ca^2+^ overload and subsequent cell death. These findings revealed that i) the exacerbated ER Ca^2+^ release in response to photodynamic stress is a novel “non-transcriptional” function of extra-nuclear p53; and ii) the cell-death resistance of p53-deficient cancer cells to photodynamic therapy is due to a failure to induce “toxic” Ca^2+^ signaling events. Excitingly, in another recent study [46], Pinton's team showed that cytosolic p53 accumulated at ER-mitochondrial contact sites in response to chemotherapeutic agents/cell-death stimuli. This event promoted ER-mitochondrial Ca^2+^ transfer and mitochondrial Ca^2+^ overload and thus apoptotic cell death. Cells lacking p53 failed to display these responses upon chemotherapy treatment. This could be restored by p53 lacking its nuclear localization sequence or by p53 targeted to the ER, but not by naturally occurring oncogenic p53 mutants. The effects of wild-type p53 on mitochondrial Ca^2+^ signaling in cells exposed to chemotherapy were found to be caused by a direct interaction of ER-localized p53 with SERCAs, lowering their oxidation state, accelerating Ca^2+^-pump activity and provoking ER Ca^2+^ overload. Oncogenic p53 mutants failed to promote SERCA activity and thus lacked the boosting of ER-mitochondrial transfers of “cytotoxic” Ca^2+^ signals. Consistent with these findings, an apoptotic response of p53-deficient cancer cells to photodynamic therapy could also be restored by overexpressing either SERCA or the MCU, thereby enhancing “toxic” Ca^2+^-signaling events. Next, the team performed *in vivo* Ca^2+^ imaging in three-dimensional tumor masses in mouse models using a skinfold chamber technique, visualizing for the first time intracellular Ca^2+^ dynamics in tumors exposed to photodynamic therapy in living animals. In these tumor masses, photodynamic stress prominently elevated cytosolic and mitochondrial [Ca^2+^] *in vivo*, resulting in tumor cell death. Buffering intracellular Ca^2+^ prevented this *in vivo* photodynamic stress-induced tumor cell death. These events critically depended on p53, since p53-deficient tumor cells failed to display photodynamic stress-induced [Ca^2+^] rise and cell death.

These elegant *in vivo* experiments underpin the central role of ER-mitochondrial Ca^2+^ transfers not only in cell death but also in therapeutic responses to anti-cancer strategies. In addition, they underscore the potential of potentiating Ca^2+^-transport systems at the level of the ER and the mitochondria to overcome cell-death resistance of tumors (like p53-deficient cancers) to therapeutic treatments. These studies also highlight the importance of considering and investing in the future application of Ca^2+^-signaling-based therapies to increase the therapeutic success of anti-cancer treatments. In particular, treatments based on inducing tumor cell death by phototherapy and likely also chemotherapy, could be boosted by activation of toxic Ca^2+^-signaling. The search for molecules that could improve ER-mitochondrial Ca^2+^ fluxes, being it activators of SERCA, IP Rs, VDAC1 or MCU, may turn out to be promising tools in patients that suffer from tumors that poorly respond to therapeutic regimens. Given the central role of Ca^2+^ signaling in a variety of physiological processes, an important challenge will be to avoid toxic effects in unaltered, healthy cells. Strategies could involve the local release of compounds using coated nanoparticles or their coupling via a protease-sensitive linker sequence to a chemical moiety. This would allow the compound to enter cancer, but not healthy, cells expressing the particular protease. This strategy has been successfully used for analogues of thapsigargin, a high-affinity, irreversible inhibitor of SERCA [47]. Further opportunities will also lie in mapping and understanding the altered “Ca^2+^-signaling” context of cancer cells due to deranged expression of Ca^2+^ channels and pumps or deranged signaling pathways involved in the activation of these channels, like arachidonic acid signaling towards plasmalemmal Orai1/Orai3 channels in prostate cancers [48] and IP_3_ signaling towards type 2 IP_3_Rs in B-cell malignancies [49]. Indeed, normal and some B-cell cancer cells appear to withstand the disruption of IP R/Bcl-2 complexes, while other B-cell cancer cells are very sensitive to this treatment [49, 50]. This indicates a critical difference between normal versus cancer cells on the one hand and between different subsets of cancer cells on the other hand in their addiction to Bcl-2 at level of their ER/IP Rs [51]. These insights may lead to the development of innovative “Ca^2+^-signaling drugs” that could enhance Ca^2+^ fluxes in (a subset of) cancer, but not in healthy, cells, thereby killing or sensitizing these cancers, including resistant p53-deficient cancers, to treatments.

In summary, these recent findings by Pinton and co-workers elucidate that the ability of chemo-/photo-therapies to trigger pro-apoptotic Ca^2+^ signaling from the ER into the mitochondria, critically depends on the presence of p53. These data reveal that pro-apoptotic Ca^2+^ signaling is an important factor that determines the success of these strategies to kill cancer cells, including those related to oncogenic p53 mutants. This work also provides good hope that promoting Ca^2+^ signaling from the ER into the mitochondria could be beneficial for the targeting of a variety of tumors, including tumors resistant to chemotherapy, and re-establishing sensitivity of malignant cells to anti-cancer therapies.
